# The role of watermelon caffeic acid *O*-methyltransferase (*ClCOMT1*) in melatonin biosynthesis and abiotic stress tolerance

**DOI:** 10.1038/s41438-021-00645-5

**Published:** 2021-10-01

**Authors:** Jingjing Chang, Yanliang Guo, Jingyi Yan, Zixing Zhang, Li Yuan, Chunhua Wei, Yong Zhang, Jianxiang Ma, Jianqiang Yang, Xian Zhang, Hao Li

**Affiliations:** 1 State Key Laboratory of Crop Stress Biology for Arid Areas, College of Horticulture, Northwest A&F University, Yangling, Shaanxi China; 2 State Key Laboratory of Vegetable Germplasm Innovation, Tianjin, China

**Keywords:** Abiotic, Transgenic plants

## Abstract

Melatonin is a pleiotropic signaling molecule that regulates plant growth and responses to various abiotic stresses. The last step of melatonin synthesis in plants can be catalyzed by caffeic acid *O*-methyltransferase (COMT), a multifunctional enzyme reported to have *N*-acetylserotonin *O*-methyltransferase (ASMT) activity; however, the ASMT activity of COMT has not yet been characterized in nonmodel plants such as watermelon (*Citrullus lanatus*). Here, a total of 16 putative *O-methyltransferase* (*ClOMT*) genes were identified in watermelon. Among them, *ClOMT03* (*Cla97C07G144540*) was considered a potential *COMT* gene (renamed *ClCOMT1*) based on its high identities (60.00–74.93%) to known *COMT* genes involved in melatonin biosynthesis, expression in almost all tissues, and upregulation under abiotic stresses. The ClCOMT1 protein was localized in the cytoplasm. Overexpression of *ClCOMT1* significantly increased melatonin contents, while *ClCOMT1* knockout using the CRISPR/Cas-9 system decreased melatonin contents in watermelon calli. These results suggest that *ClCOMT1* plays an essential role in melatonin biosynthesis in watermelon. In addition, *ClCOMT1* expression in watermelon was upregulated by cold, drought, and salt stress, accompanied by increases in melatonin contents. Overexpression of *ClCOMT1* enhanced transgenic *Arabidopsis* tolerance against such abiotic stresses, indicating that *ClCOMT1* is a positive regulator of plant tolerance to abiotic stresses.

## Introduction

Melatonin (*N*-acetyl-5-methoxytryptamine), an important bioactive molecule, is ubiquitously present in living organisms throughout the animal, plant, and many other kingdoms^1,2^. Melatonin was identified in vascular plants in 1995;^3,4^ since then, numerous studies have proven the multiple functions of melatonin in plant growth and development, postharvest physiology, and adaptation to various environmental stresses, such as cold, salinity, drought, and pathogens^5–7^. Recently, the discovery of the first melatonin receptor (CAND2/PMTR1) in *Arabidopsis* provided strong evidence for considering melatonin as a new plant hormone^8,9^.

The quantities of melatonin in plant cells vary considerably among species, ranging from several picograms to a few micrograms per gram of fresh tissue^10^. Melatonin in plants is synthesized from tryptophan via four consecutive enzymatic steps^6,11,12^. In the first two steps, tryptophan is converted into serotonin by tryptophan decarboxylase, tryptophan hydroxylase, and tryptamine 5-hydroxylase. In the final two steps, serotonin is catalyzed into *N*-acetylserotonin (NAS) by serotonin *N*-acetyltransferase (SNAT) in chloroplasts, and NAS is subsequently methylated into melatonin by *N*-acetylserotonin *O*-methyltransferase (ASMT) in the cytoplasm^13^. Some studies suggest that ASMT, as the terminal enzyme, plays a rate-limiting role in melatonin biosynthesis in plants^6,14^.

The first *ASMT* gene (*OsASMT1*) was cloned from the monocotyledonous species rice (*Oryza sativa*) in 2011^15^. In addition to *OsASMT1*, overexpression of *OsASMT2* and *OsASMT3* in rice calli also enhanced melatonin production^16^. Subsequent studies cloned the *ASMT* genes from dicotyledonous plant species, including *Arabidopsis*, *Malus zumi*, and *Hypericum perforatum*^17–19^. However, the homologies of *ASMT* genes vary considerably among plant species. The homologous genes of rice *ASMT* are found only in some other monocotyledonous plants^15,16^. *ASMT* genes from dicotyledonous *Arabidopsis* and *Malus zumi* encoded proteins that share only 31% and 40% identity, respectively, with *OsASMT1*^17,18^. In addition to ASMT, caffeic acid *O*-methyltransferase (COMT), a multifunctional enzyme, was also reported to have ASMT activity^20,21^. Based on enzyme catalysis, both ASMT and COMT belong to the *O*-methyltransferase (OMT) family, which methylates a variety of secondary metabolites, such as phenylpropanoids, alkaloids, and flavonoids^20,22^. *COMT* genes have been identified in various plant species, such as *Lolium perenne*, *Chrysanthemum grandiflorum*, *Hordeum vulgare*, *Brachypodium distachyon*, and *Populus tremuloides*^23–29^. However, their involvement in melatonin biosynthesis was reported only in model plants, including monocotyledonous rice and dicotyledonous *Arabidopsis* and *Solanum lycopersicum*^20,21,30^. Melatonin biosynthesis-related *COMT* genes from other plant species need to be further identified to exploit their new functions. Moreover, *COMT* genes with *ASMT* function from crops can be utilized for enhancement of crop yield and quality through genetic manipulation and rational breeding.

Watermelon (*Citrullus lanatus* (Thunb.) Matsum. & Nakai), one of the top five most consumed fresh fruits, is widely grown as an important horticultural crop worldwide. The cultivated area of watermelon in 2018 was 3.24 million hectares worldwide (http://www.fao.org/). Previous investigators have reported the positive effects of exogenous melatonin on watermelon tolerance against abiotic stresses, including cold, salt, drought, and vanadium stress;^31–34^ however, the melatonin biosynthetic genes in watermelon have not been cloned and characterized due to the relatively difficult process of genetic transformation in watermelon. Here, we performed bioinformatics analysis of watermelon *ClOMT* genes, cloned a potential *COMT* gene (renamed *ClCOMT1*), and further characterized its function in melatonin production via overexpression or knockout of *ClCOMT1* in watermelon calli. In addition, we investigated the role of *ClCOMT1* in tolerance against abiotic stresses, including cold, drought, and salinity, by overexpressing *ClCOMT1* in *Arabidopsis*.

## Materials and methods

### Identification of *O-methyltransferase* (*OMT*) genes in watermelon

The watermelon protein database (watermelon_v2.pep) was obtained from the Cucurbit Genomics Database (CuGenDB, http://cucurbitgenomics.org/)^35^. Then, the hidden markov model (HMM) profile of the *O*-methyltransferase domain (PF00891) from Pfam (http://pfam.xfam.org/) was utilized to identify *OMT* genes from the watermelon protein database with an e-value < 1 × e^−10^. The presence of the PF00891 domain in all the protein sequences obtained was analyzed using the SMART tool (http://smart.embl-heidelberg.de/)^36^.

### Chromosomal locations and phylogenetic analysis

The chromosomal location image of *ClOMT*s was drawn using MapChart2.32^37^, on the basis of chromosomal position information from the watermelon genome website (http://cucurbitgenomics.org/organism/21). Tandem duplicated genes were identified based on two criteria: (a) adjacent homologous genes were located on the same chromosome with no more than one intervening gene, and (b) the length and similarity of aligned sequences were >70%^38,39^. Multiple sequence alignments of ClOMT protein sequences were carried out via ClustalW with default parameters^40^. Based on the alignment results, a phylogenetic tree was constructed in MEGA 7.0.21 software by using the neighbor-joining method with 1000 bootstrap replications and a Jones-Taylor-Thornton (JTT) matrix-based model^41^.

### Expression analysis of *ClOMT* genes based on mRNA sequencing

The expression of watermelon *ClOMT* genes in different tissues and in response to abiotic stresses was analyzed based on mRNA sequencing data from CuGenDB (http://cucurbitgenomics.org/). The tested tissues included fruit flesh, fruit rind 10, 18, 26, and 34 days after pollination, seeds 49 days after pollination, phloem, vascular bundle, root, and leaf. For abiotic stress treatments, the watermelon seedlings were exposed to 4 °C for 6 h, water was withheld for eight days, or PEG 6000 (20%) was applied to the roots for 6 h. Leaf tissues were harvested after cold and drought, while root samples were taken after PEG 6000 treatment. Transcript levels were calculated as reads per kilobase of exon model per million mapped reads (RPKM). The RPKM values were log2 transformed for tissue-specific analysis, while the RPKM ratio of Treatment/Control was log2 transformed for stress response analysis. The heat maps were drawn using Multiple Experiment Viewer (Version Mev4.9)^42^.

### Subcellular localization of ClCOMT1

The pGREEN vector fused with green fluorescent protein (GFP) was used to determine the subcellular localization of the ClCOMT1 protein. The cDNA sequence of *ClCOMT1* was PCR-amplified with primers containing *EcoR*V and *Xho*I restriction sites. The amplified products were gel-purified and ligated into the pGREEN vector. Then, the recombinant plasmid pGREEN-*ClCOMT1*-GFP was transiently transformed into watermelon leaf protoplasts. Protoplast extraction and transfection were performed as described previously using 20-day-old watermelon leaves^43^. Fluorescence was observed using a confocal laser scanning microscope (Leica TCS-SP8 SR, Germany).

### Vector construction and gene transformation in watermelon calli

To knock out *ClCOMT1* in wild-type watermelon calli, the CRISPR/Cas9 construct was generated using the binary vector PBSE402 with two guide RNAs designed by CRISPR-P v2.0 (http://crispr.hzau.edu.cn/CRISPR2/)^44,45^. Overexpression of *ClCOMT1* in watermelon calli was completed using the pCambia1305.4 vector. A 1074-bp *ClCOMT1* cDNA was PCR-amplified with specific primers harboring *BamH*I and *Pml*I restriction sites and was then ligated into the pCambia1305.4 vector. Recombinant PBSE402-*ClCOMT1* or pCambia1305.4-*ClCOMT1* was transformed into watermelon by *Agrobacterium tumefaciens-*mediated transformation (strain EHA105)^43^. A potentially successful transformation was detected by monitoring GFP fluorescence under a stereoscopic fluorescence microscope (MZ10F, Leica, Germany). Genomic DNA fragments with target sites were amplified to determine the mutation types using high-throughput tracking of mutations (Hi-TOM)^46^. Expression of *ClCOMT1* in the transgenic or wild-type calli was analyzed by qRT-PCR. Specific amplification primers are given in Supplementary Table S1.

### Transgenic *Arabidopsis* plant generation and abiotic stress tolerance assay

To overexpress *ClCOMT1* in *Arabidopsis*, the recombinant plasmid pGREEN-*ClCOMT1*-GFP was transformed into *Agrobacterium tumefaciens* (strain GV3101), which was then transformed into *Arabidopsis* using the floral dip method^47^. Transgenic *Arabidopsis* plants were screened by BASTA. Transcripts of *ClCOMT1* were determined in the leaves of four-week-old wild-type or T_0_ transgenic *Arabidopsis* using semiquantitative RT-PCR. Detailed primer sequences are given in Supplementary Table S1.

Homozygous T_3_ transgenic line #4 was used for analysis of abiotic stress tolerance. After vernalization at 4 °C for 3 d, transgenic and wild-type *Arabidopsis* seeds were cultured on half-strength Murashige and Skoog (MS) plates in a growth chamber at 22 °C. Freezing stress treatment was carried out as described by Hu et al. with a slight modification^48^. Two-week-old seedlings were exposed to −10 °C for 1 h and recovered at 22 °C for 4 d. For drought and salt stress treatments, the vernalized seeds were sown and grown in ½ MS containing 250 mM D-mannitol and 75 mM NaCl for 18 days, respectively^18,49^. Survival rates of *Arabidopsis* seedlings were recorded.

### Quantitative RT-PCR and semiquantitative RT-PCR

Total RNA was extracted using the RNA simple Total RNA Kit (TIANGEN, Beijing, China) following the manufacturer’s instructions. One microgram of total RNA was reverse-transcribed using the FastKing RT Kit with gDNase (TIANGEN, Beijing, China). The qRT-PCR assay was conducted by a StepOnePlus^TM^ Real-Time PCR System (Applied Biosystems, USA). PCR assays were carried out using the SYBR^®^ Premix ExTaq^TM^ II (2×) kit (Takara, Tokyo, Japan). The relative expression of mRNA was quantified via normalization to watermelon *β-actin*^50^, and calculated as described previously^51^. For the semiquantitative RT-PCR assay, PCR-amplified products were electrophoresed on 1% TAE-agarose gels. The gene-specific primers are shown in Supplementary Table S1.

### Melatonin assay

Melatonin was extracted using the acetone–methanol method^52^ and measured using an ELISA kit (Shanghai Lanpai Biotechnology Co., Ltd, Shanghai, China) according to the manufacturer’s instructions. Colorimetric recording was carried out via a Multimode Plate Reader M200 pro (Tecan, Männedorf, Switzerland).

### Cold, drought, and salt treatment of watermelon

Watermelon (cv. Nongkeda No. 5) seedlings were cultured in a greenhouse at Northwest Agriculture and Forestry University, Yangling, China. Seedlings with three true leaves were treated with cold at 4 °C, unwatered, or irrigated with NaCl solution (300 mM, 80 mL per plant). Leaf samples were taken after cold, drought, or salt treatment for 24 h, 4 d, or 2 d, respectively.

### Statistical analysis

All data were analyzed with SPSS software by using Student’s *t* test, and *P* < 0.05 was considered to indicate statistical significance.

## Results

### Bioinformatics analysis and expression profiles of the *ClOMT* genes in watermelon

A total of 16 putative *ClOMT* genes were identified in watermelon using the HMM search program and were renamed *ClOMT01* to *ClOMT16* based on their gene ID in the watermelon reference genome (Table 1). Among ClOMT proteins, ClOMT03 was annotated as caffeic acid 3-*O*-methyltransferase 1, while ClOMT01 and ClOMT02 were annotated as caffeic acid 3-*O*-methyltransferase-like.

**Table 1:** *O-methyltransferase* genes in watermelon

Gene Name	Gene ID	Chromosome location	Description
*ClOMT01*	*Cla97C10G188660*	Chr10: 4750672..4753331	caffeic acid 3-*O*-methyltransferase-like
*ClOMT02*	*Cla97C10G188670*	Chr10: 4767376..4770041	caffeic acid 3-*O*-methyltransferase-like
*ClOMT03*	*Cla97C07G144540*	Chr07: 31874671..31875744	caffeic acid 3-*O*-methyltransferase 1
*ClOMT04*	*Cla97C02G043210*	Chr02: 31427713..31428863	*O*-methyltransferase, family 2
*ClOMT05*	*Cla97C02G043200*	Chr02: 31402942..31404092	*O*-methyltransferase, family 2
*ClOMT06*	*Cla97C09G172390*	Chr09: 8789656..8791046	*O*-methyltransferase, family 2
*ClOMT07*	*Cla97C02G028680*	Chr02: 2161556..2164053	*O*-methyltransferase, family 2
*ClOMT08*	*Cla97C02G030510*	Chr02: 3482833..3484720	*O*-methyltransferase, family 2
*ClOMT09*	*Cla97C02G030520*	Chr02: 3488829..3491274	*O*-methyltransferase, family 2
*ClOMT10*	*Cla97C02G030540*	Chr02: 3508413..3510435	*O*-methyltransferase, family 2
*ClOMT11*	*Cla97C02G030550*	Chr02: 3515980..3518336	*O*-methyltransferase, family 2
*ClOMT12*	*Cla97C02G030560*	Chr02: 3542077..3543859	*O*-methyltransferase, family 2
*ClOMT13*	*Cla97C02G030570*	Chr02: 3547673..3554475	*O*-methyltransferase, family 2
*ClOMT14*	*Cla97C10G195930*	Chr10: 25518757..25520719	*O*-methyltransferase
*ClOMT15*	*Cla97C10G195920*	Chr10: 25509231..25511161	*O*-methyltransferase
*ClOMT16*	*Cla97C10G202790*	Chr10: 32622705..32628072	*O*-methyltransferase

The gene description sourced from the CuGenDB (http://cucurbitgenomics.org/). *ClOMT Citrullus lanatus O-methyltransferase*

**Fig. 1:**
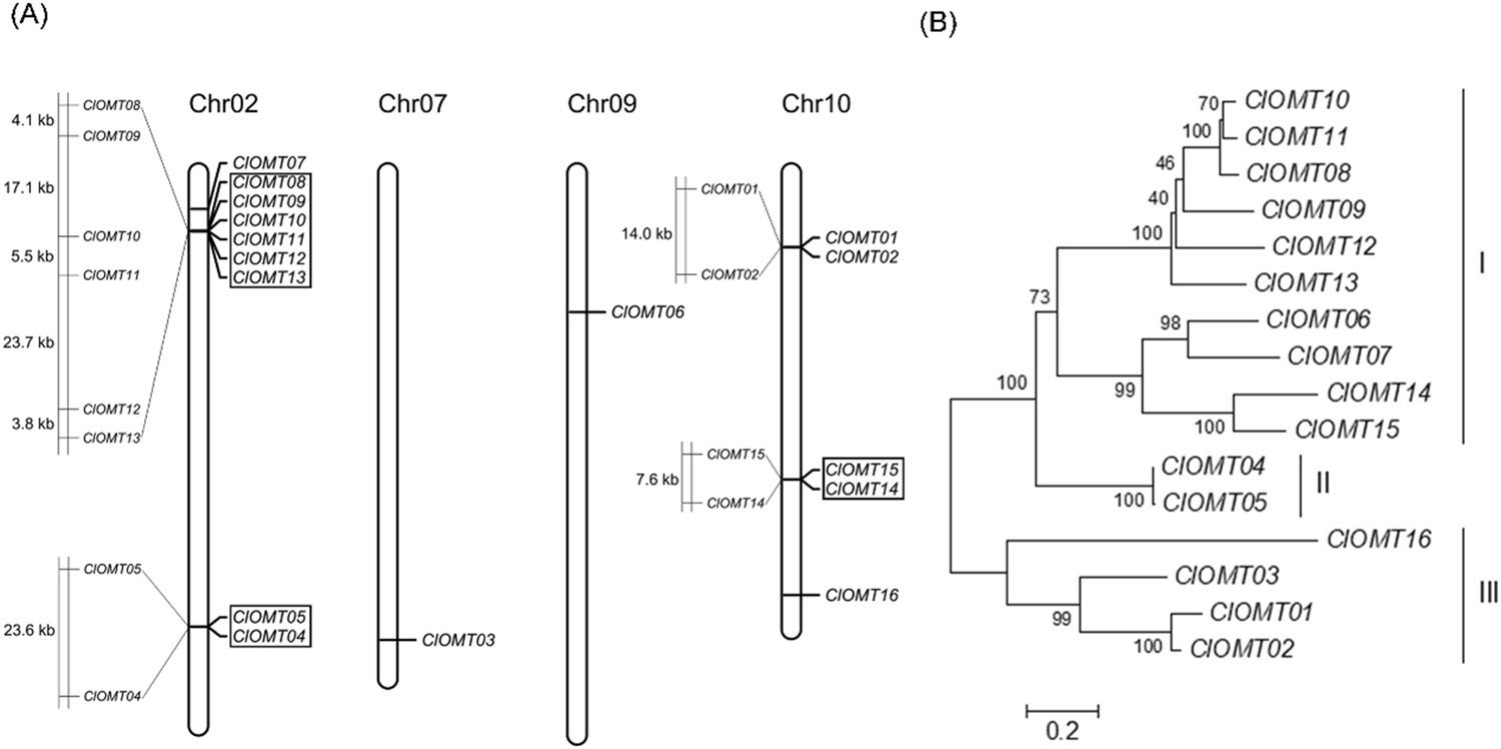
Chromosomal locations and phylogenetic analysis of ClOMT genes in watermelon. **A** The chromosomal location was drawn using MapChart2.32. The tandem duplicated genes are boxed. **B** The phylogenetic tree of the *ClOMT* genes was constructed using MEGA 7.0.21, and the *ClOMT* genes were placed into groups I, II and III. Bar, 0.2 substitutions per site; Chr, chromosome; *ClOMT Citrullus lanatus O-methyltransferase*

The 16 *ClOMT* genes were unevenly distributed on chromosomes 02, 07, 09 and 10 in the watermelon genome (Fig. 1A). Some *ClOMTs* mapped to neighboring regions on the same chromosome. According to the defined criteria^38,39^, *ClOMT01*, *ClOMT02*, *ClOMT03*, *ClOMT06*, *ClOMT07*, and *ClOMT16* were not tandemly duplicated genes, while the other *ClOMT* genes seemed to be produced from tandem duplications. As shown in Fig. 1B, *ClOMTs* were classified into three groups (I–III) according to phylogenetic analyses, with ten *ClOMTs* in group I, two in group II (*ClOMT04* and *ClOMT05*), and four in group III (*ClOMT01*, *ClOMT02*, *ClOMT03* and *ClOMT16*).

**Fig. 2:**
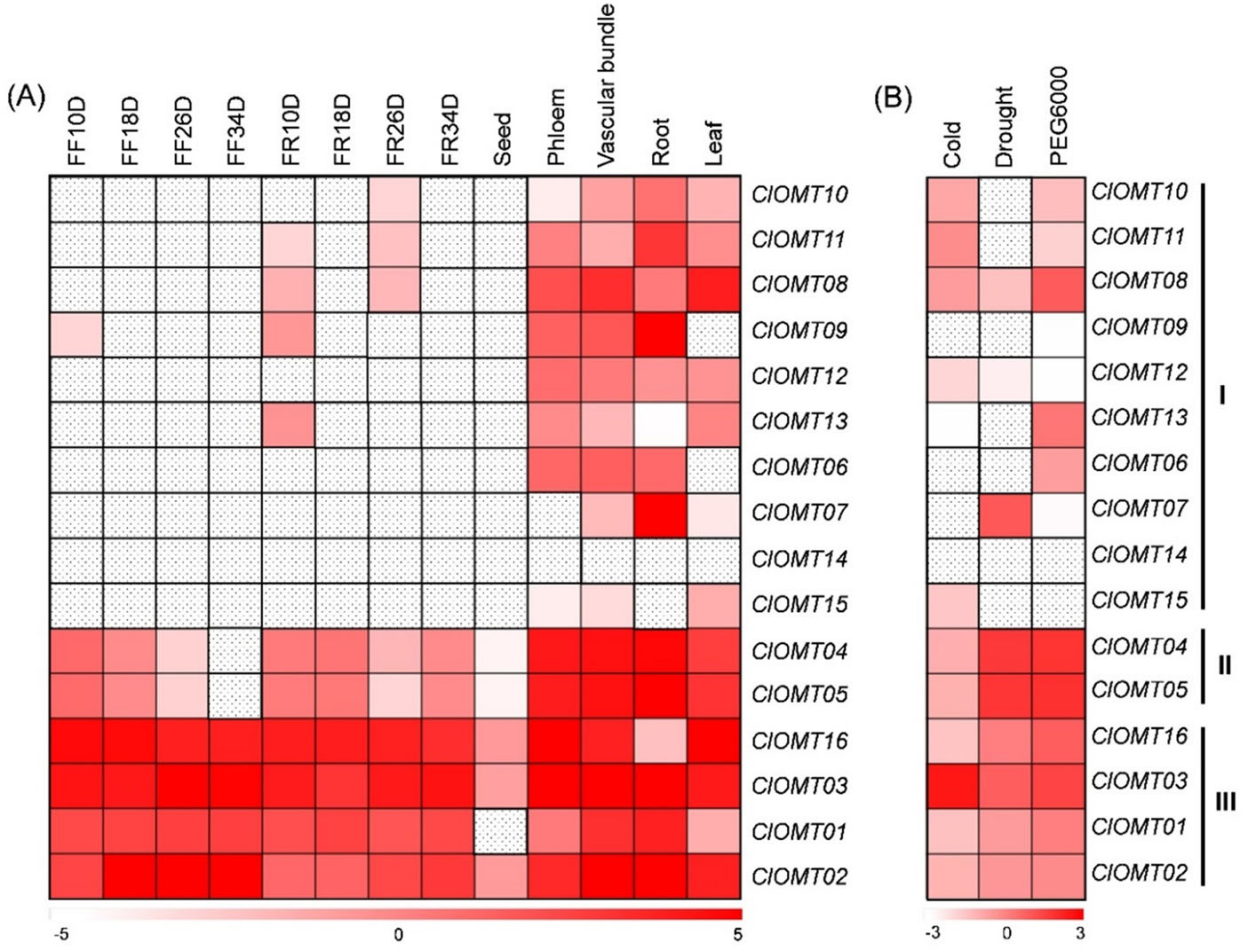
The expression of ClOMT genes in different tissues and in response to abiotic stresses. Heat maps of *ClOMT* genes in (**A**) different tissues and (**B**) in response to cold, drought, or osmotic stress. The RNA-seq data were obtained from the CuGenDB (http://cucurbitgenomics.org/). Color coding was done according to the scale given. Boxes filled with dots indicate no detection. In (**A**), watermelon tissues included fruit flesh (FF), fruit rind (FR) 10, 18, 26, and 34 days after pollination, seeds 49 days after pollination, phloem, vascular bundle, root, and leaf tissues. Transcript levels were calculated as log2-transformed Reads Per Kilobase of exon model per Million mapped reads (RPKM). In (**B**), the watermelon seedlings were exposed to 4 °C for 6 h, unwatered for eight days, or treated with PEG 6000 (20%) on roots for 6 h. Leaf tissues were harvested after cold and drought, while root samples were taken after PEG 6000 treatment. The values were calculated as log2-transformed RPKM ratios of Treatment/Control. *ClOMT Citrullus lanatus O-methyltransferase*

The temporal and spatial expression of *ClOMT* genes was analyzed based on the RNA-seq data from CuGenDB (http://cucurbitgenomics.org/). Only *ClOMT02*, *ClOMT03* and *ClOMT16* were constitutively expressed in all watermelon tissues tested, including fruit flesh, fruit rind, seed, phloem, vascular bundle, root, and leaf tissues (Fig. 2A). The *ClOMT01* gene was undetected only in seeds, while *ClOMT04* and *ClOMT05* were undetected only in fruit flesh 34 days after pollination. Most *ClOMT* genes in group I were not detected in fruit flesh, fruit rind, or seeds. Expression of *ClOMT14* was not observed in any of the tissues tested.

The production of melatonin can be induced by various abiotic stresses^53^. We further analyzed the expression of *ClOMT* genes in response to abiotic stresses, including cold, drought, and osmotic stress, simulated using 20% PEG 6000. The *ClOMT* genes exhibited different expression patterns in response to different stresses (Fig. 2B). Only *ClOMT03* expression was upregulated by all three stresses. The expression of *ClOMT04* and *ClOMT05* was upregulated under both drought and osmotic stress. The transcript levels of *ClOMT08* and *ClOMT16* were induced only by osmotic stress, while that of *ClOMT07* was induced only by drought.

### Sequence analysis and subcellular localization of the ClCOMT1 protein

To further narrow the range of candidate *COMT* genes, we analyzed the sequence identities of *ClOMT01*, *ClOMT02*, *ClOMT03* and *ClOMT16* to the *ASMTs* and melatonin biosynthesis-related *COMTs* reported in other plant species (Table 2). These four *ClOMT*s had <43% identity to *ASMT*s in *Arabidopsis*, *Malus zumi*, and rice. The *ClOMT03* gene exhibited the highest identities to *COMT*s in *Arabidopsis* (74.93%), tomato (74.30%), and rice (60.00%). Based on the above results, *ClOMT03* was considered a potential watermelon *COMT* gene involved in melatonin biosynthesis and was renamed *ClCOMT1*.

**Table 2:** The homology of *ClOMT01*, *ClOMT02*, *ClOMT03*, and *ClOMT16* to *ASMT*/*COMT* genes from other plant species

Gene Name	*COMT*			*ASMT*		
	*AtCOMT*	*SlCOMT1*	*OsCOMT*	*AtASMT*	*MzASMT1*	*OsASMT1*
*ClOMT01*	50.31%	52.30%	46.20%	42.86%	29.00%	26.45%
*ClOMT02*	54.52%	56.73%	50.68%	34.12%	33.53%	30.59%
*ClOMT03*	74.93%	74.30%	60.00%	33.13%	32.09%	28.21%
*ClOMT16*	36.48%	34.03%	32.63%	28.16%	26.24%	28.37%

*AtCOMT*, *AT5G54160*; *SlCOMT1*, *Solyc03g080180*; *OsCOMT*, *LOC_Os08g06100*; *AtASMT*, *AT4G35160*; *MzASMT1*, *MD05G1308800*; *OsASMT1*, *LOC_Os09g17560*

*AtArabidopsis thaliana*, *ClCitrullus lanatus*, *MzMalus zumi*, *OsOryza sativa*, *SlSolanum lycopersicum*, *ASMTN-acetylserotonin O-methyltransferase*, *COMTcaffeic acid O-methyltransferase*, *OMTO-methyltransferase*

**Fig. 3:**
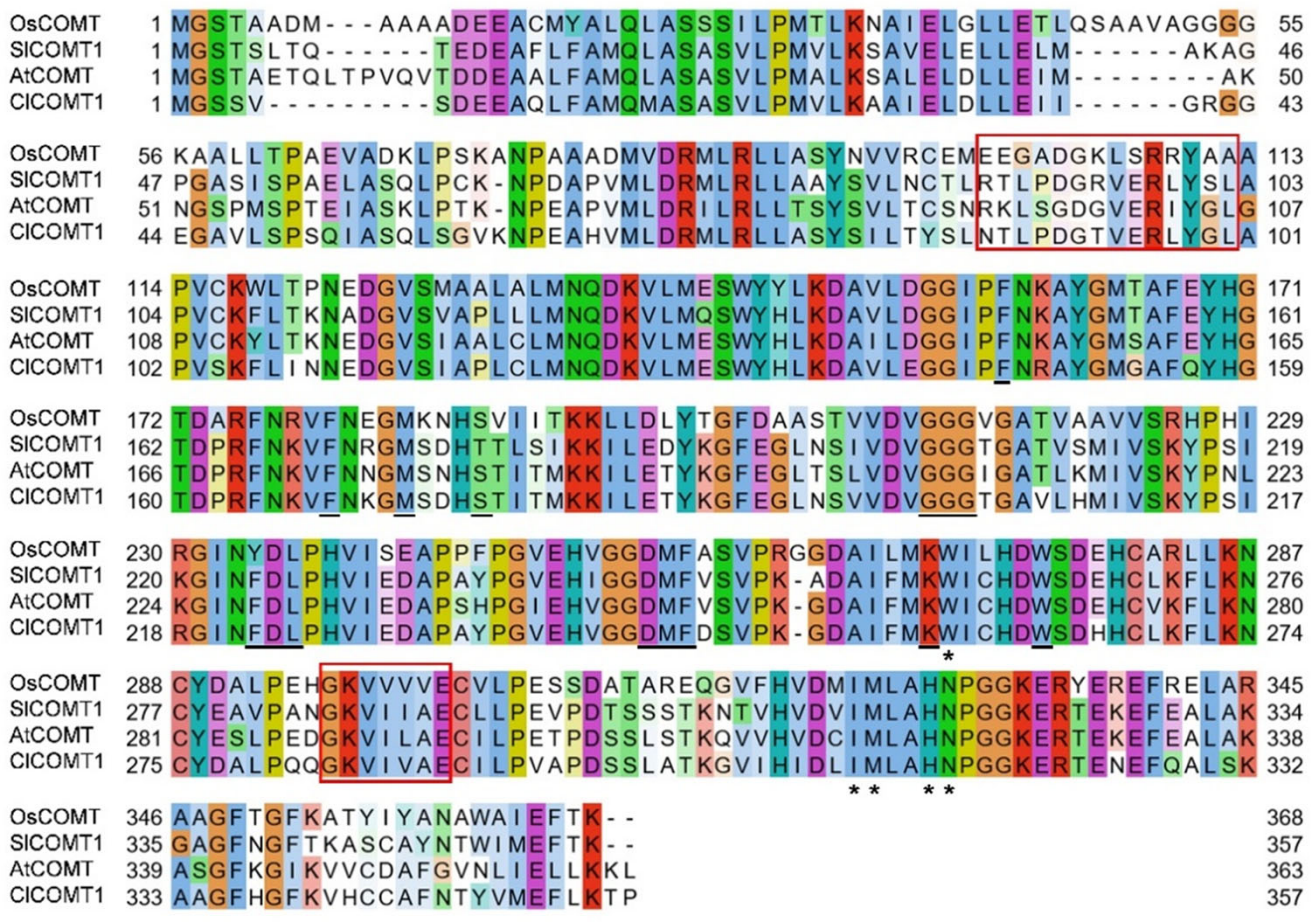
Comparison of the ClCOMT1 protein sequence with those of AtCOMT, SlCOMT1, and OsCOMT. The putative *N*-acetylserotonin (NAS)-binding domains are shown in the red box. The phenolic substrate-binding sites are marked with asterisks. The *S*-adenosyl-L-methionine (SAM)-binding sites are underlined. At Arabidopsis thaliana, Cl Citrullus lanatus, Os Oryza sativa, Sl Solanum lycopersicum, *COMT caffeic acid O-methyltransferase*

**Fig. 4:**
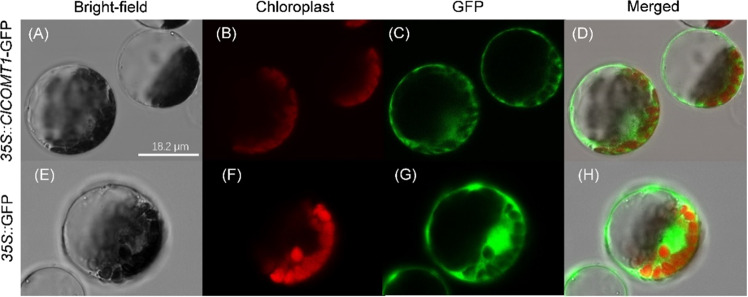
Subcellular localization of ClCOMT1 in watermelon protoplasts. **A**, **E** Bright-field image of watermelon protoplasts. **B**, **F** Red fluorescence of chlorophyll autofluorescence. **C**, **G** Green fluorescence of GFP. **D**, **H** Merged view of two fluorescence images (B + C, F + G). Bars = 18.2 µm. *ClCOMT Citrullus lanatus caffeic acid O-methyltransferase, GFP green fluorescent protein*

Like the known COMT proteins from dicotyledonous *Arabidopsis* and tomato, ClCOMT1 contained relatively conserved NAS-binding domains, phenolic substrate-binding residues, and *S*-adenosyl-L-methionine-binding sites (Fig. 3). However, the NAS-binding domains varied considerably between COMT proteins from dicotyledonous watermelon and monocotyledonous rice. The COMT proteins in rice and tomato are known to be localized in the cytoplasm^21,30^. To determine whether ClCOMT1 is also localized in the cytoplasm, the subcellular localization of ClCOMT1 in watermelon was investigated using the pGREEN-*ClCOMT1*-GFP vector. As shown in Fig. 4, green fluorescence was observed in the cytoplasm, indicating the cytoplasmic localization of ClCOMT1. The green fluorescence did not overlap with the red fluorescence of chlorophyll autofluorescence, suggesting that ClCOMT1 was not translocated into chloroplasts.

### Effects of *ClCOMT1* gene editing or overexpression on endogenous melatonin contents in watermelon calli

**Fig. 5:**
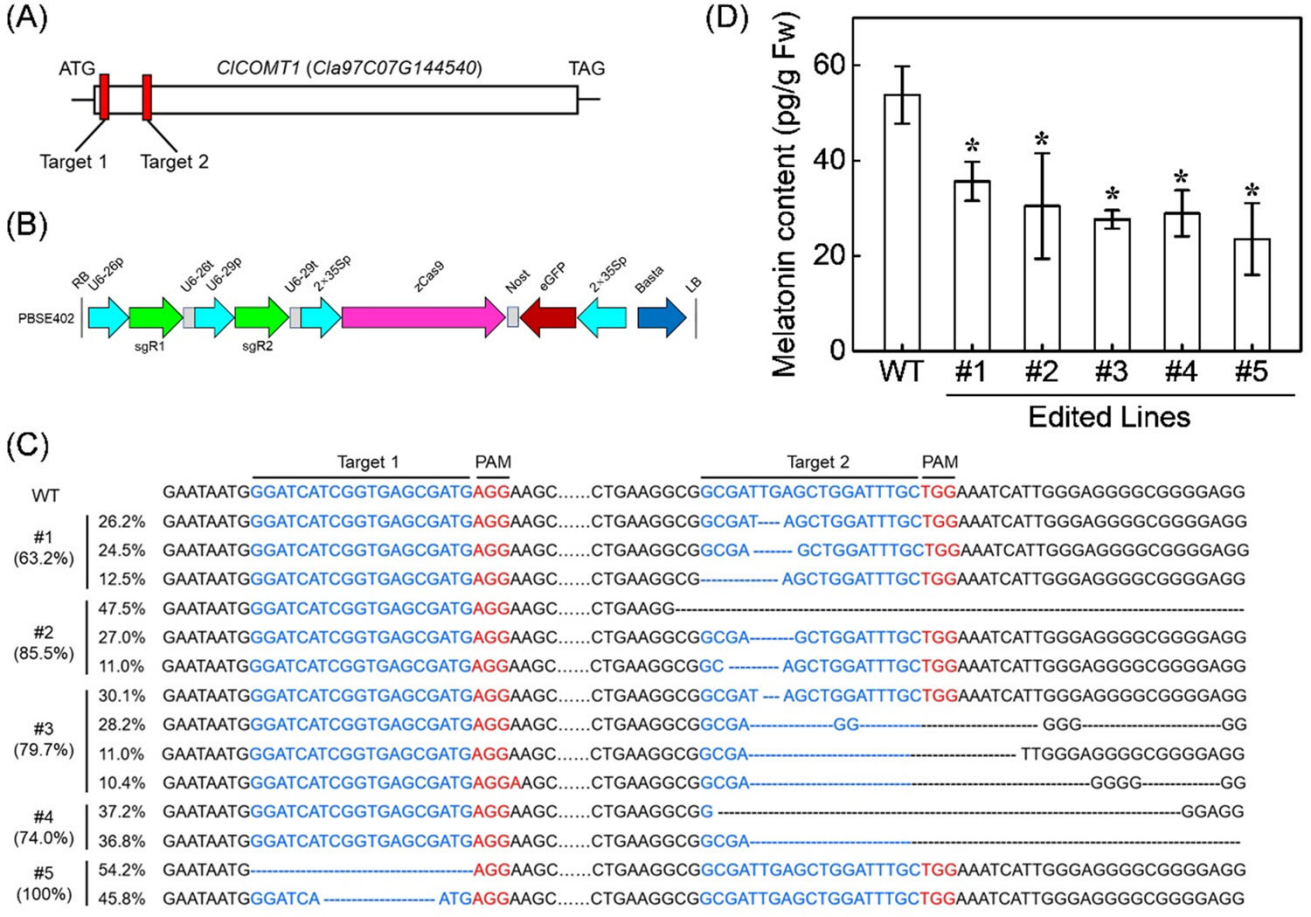
Effects of ClCOMT1 gene-editing on endogenous melatonin contents in watermelon calli. **A** Two sgRNAs (Target 1 and Target 2) in the 5’-region of *ClCOMT1*. **B** Schematic diagram for constructing two sgRNA cassettes in the binary vector PBSE402. **C** Mutation types and rates in the transgenic watermelon calli after CRISPR/Cas9-mediated gene editing. **D** Melatonin contents in transgenic and wild-type (WT) calli. In (**D**), values are means ± SD (*n* = 3). Asterisk (*) indicates significant difference at *P* < 0.05. *ClCOMT Citrullus lanatus caffeic acid O-methyltransferase*

To ascertain the direct involvement of *ClCOMT1* in melatonin biosynthesis, transgenic watermelon calli with either knockout or overexpression of *ClCOMT1* were generated. Knockout of *ClCOMT1* in watermelon calli was performed using the CRISPR/Cas9 system. Two target sites on the 5’-region of the *ClCOMT1* gene (target 1 and target 2) were assembled into the CRISPR/Cas9 vector PBSE402 (Fig. 5A, B). After transformation, five edited lines were obtained. Four lines (#1–#4) had mutations in the target 2 site, and one line (#5) had mutations in the target 1 site (Fig. 5C). Line #5 had the highest mutation rate (100%), followed by #2 (85.5%), #3 (79.7%), #4 (74.0%), and #1 (63.2%). All lines were heterozygous with 2–4 mutation types for each. For example, #5 had two mutation types (−19, −10) in target 1, whereas #3 had four (−2, −35, −24, −36) in target 2. Mutation of *ClCOMT1* resulted in a significant decrease in melatonin content (Fig. 5D). Melatonin contents in edited lines were 33.7–56.3% lower than those in wild-type calli. Moreover, melatonin content was negatively correlated with the mutation rate (Fig. S1).

**Fig. 6:**
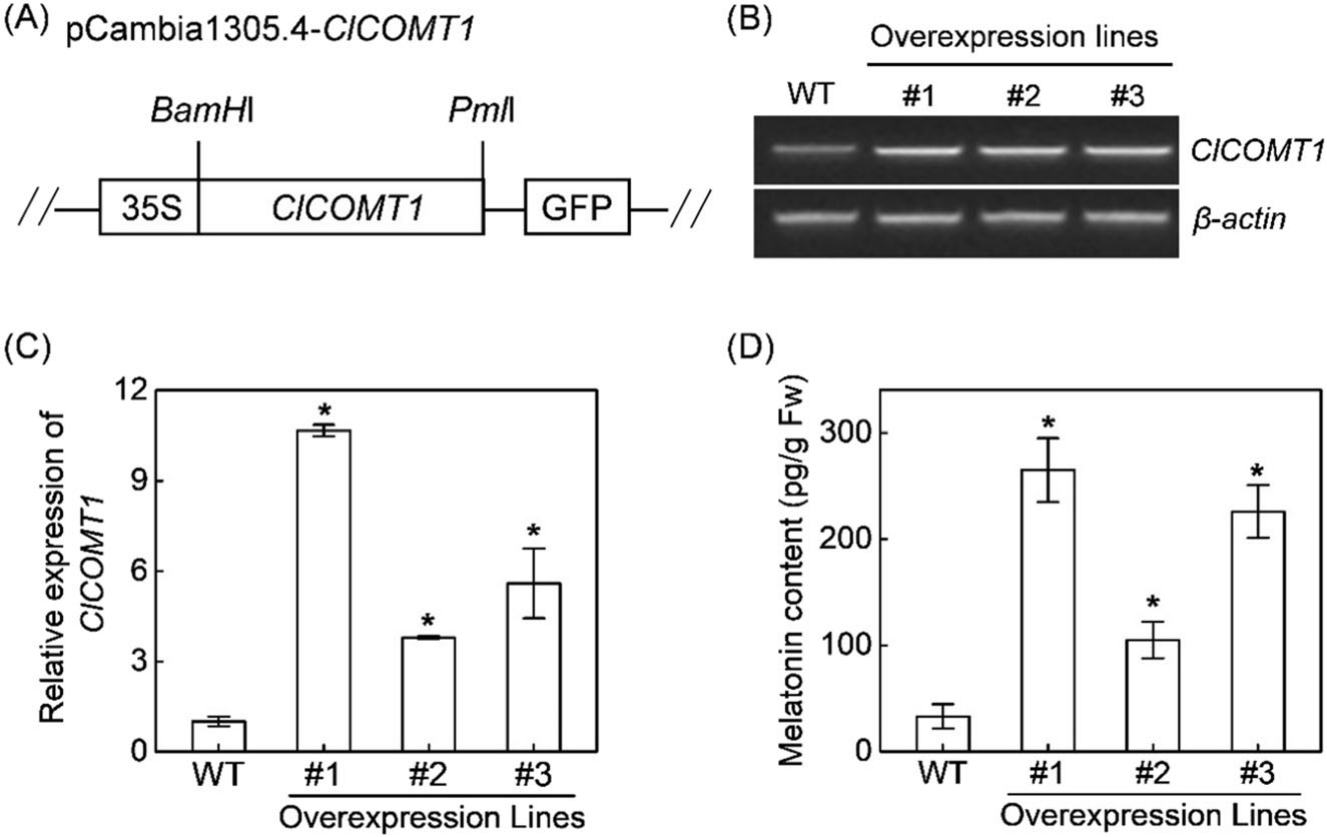
Effects of ClCOMT1 overexpression on endogenous melatonin contents in watermelon calli. **A** Schematic diagram of the pCambia1305.4-*ClCOMT1* vector. **B**, **C** RT-PCR and qRT-PCR analysis of *ClCOMT1* transcripts in overexpression lines and wild-type (WT) calli. **D** Melatonin contents in overexpression lines and WT calli. *β-actin*, watermelon internal reference gene. In (**C**, **D**), values are means ± SD (*n* = 3). Asterisk (*) indicates significant difference at *P* < 0.05. *ClCOMT Citrullus lanatus caffeic acid O-methyltransferase*

In addition, *ClCOMT1* driven by the 35 S promoter was genetically transformed into watermelon calli, leading to the overexpression of *ClCOMT1* (Fig. 6A–C). The transcript levels of *ClCOMT1* in transgenic lines #1, #2, and #3 were induced by 10.7-, 3.8-, and 5.6-fold relative to those in nontransgenic calli, respectively. Consequently, the contents of melatonin in #1, #2, and #3 increased by 7.0-, 2.2-, and 5.8-fold compared to that in wild-type calli (Fig. 6D). These results indicated a direct role of *ClCOMT1* in melatonin production in watermelon.

### The involvement of *ClCOMT1* in abiotic stresses

**Fig. 7:**
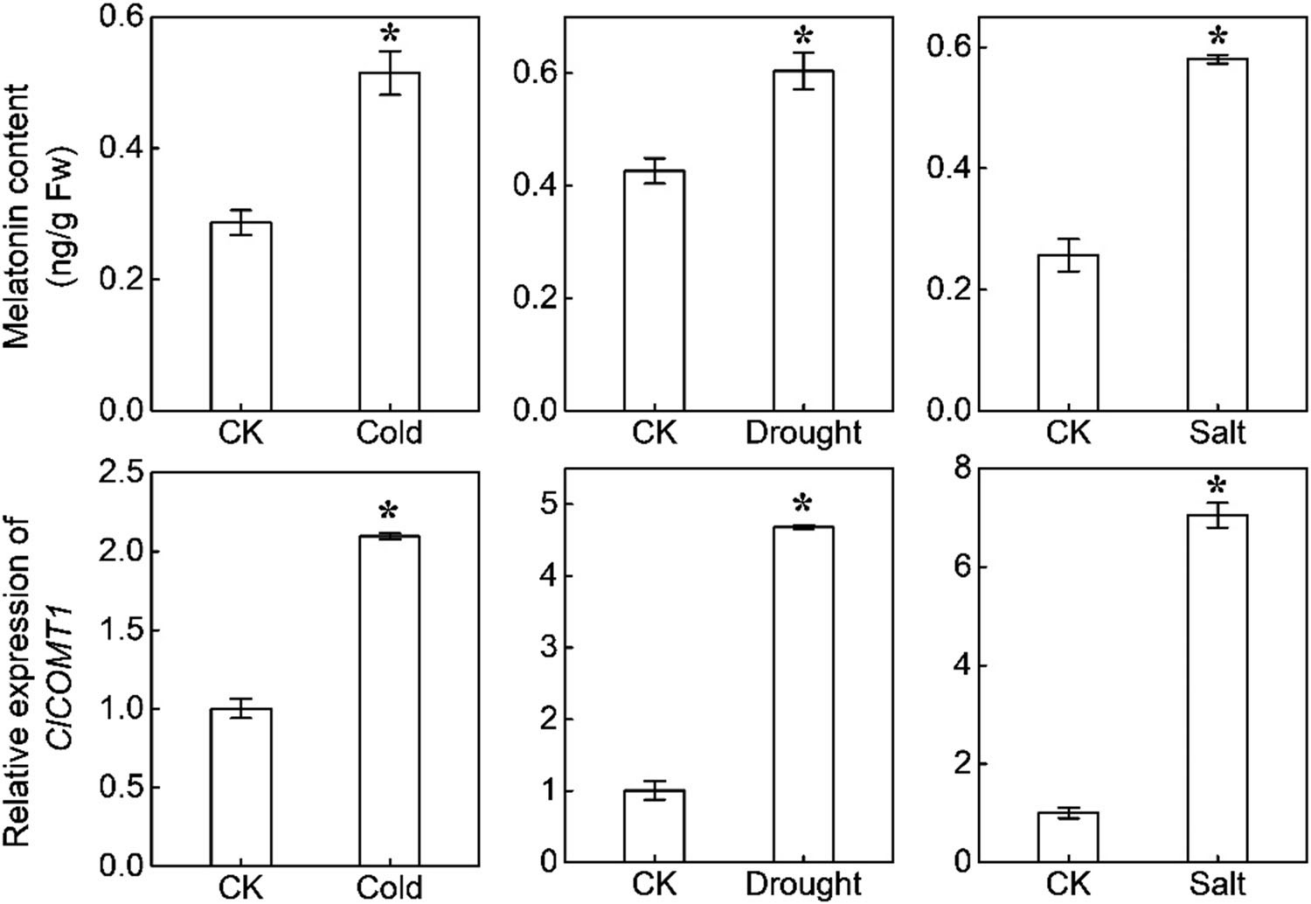
Melatonin contents and ClCOMT1 expression in response to cold, drought, or salt stress. Watermelon seedlings with three true leaves were exposed to cold at 4 °C, unwatered, or irrigated with 300 mM NaCl (80 mL per plant). Leaf samples were taken after cold, drought, or salt treatment for 24 h, 4 d, or 2 d, respectively. Values are means ± SD (*n* = 3). An asterisk (*) indicates significant difference at *P* < 0.05. CK control check, *ClCOMT*, *Citrullus lanatus caffeic acid O-methyltransferase*

The application of exogenous melatonin enhanced watermelon tolerance against cold, drought, and salt stress, as assessed through the plant phenotypes and relative electrical conductivity (Fig. S2). After exposure to these three stresses, *ClCOMT1* transcripts were induced by 2.1-, 4.7-, and 7.0-fold, and the melatonin contents were increased by 82.1%, 41.7%, and 125.9%, respectively (Fig. 7).

**Fig. 8:**
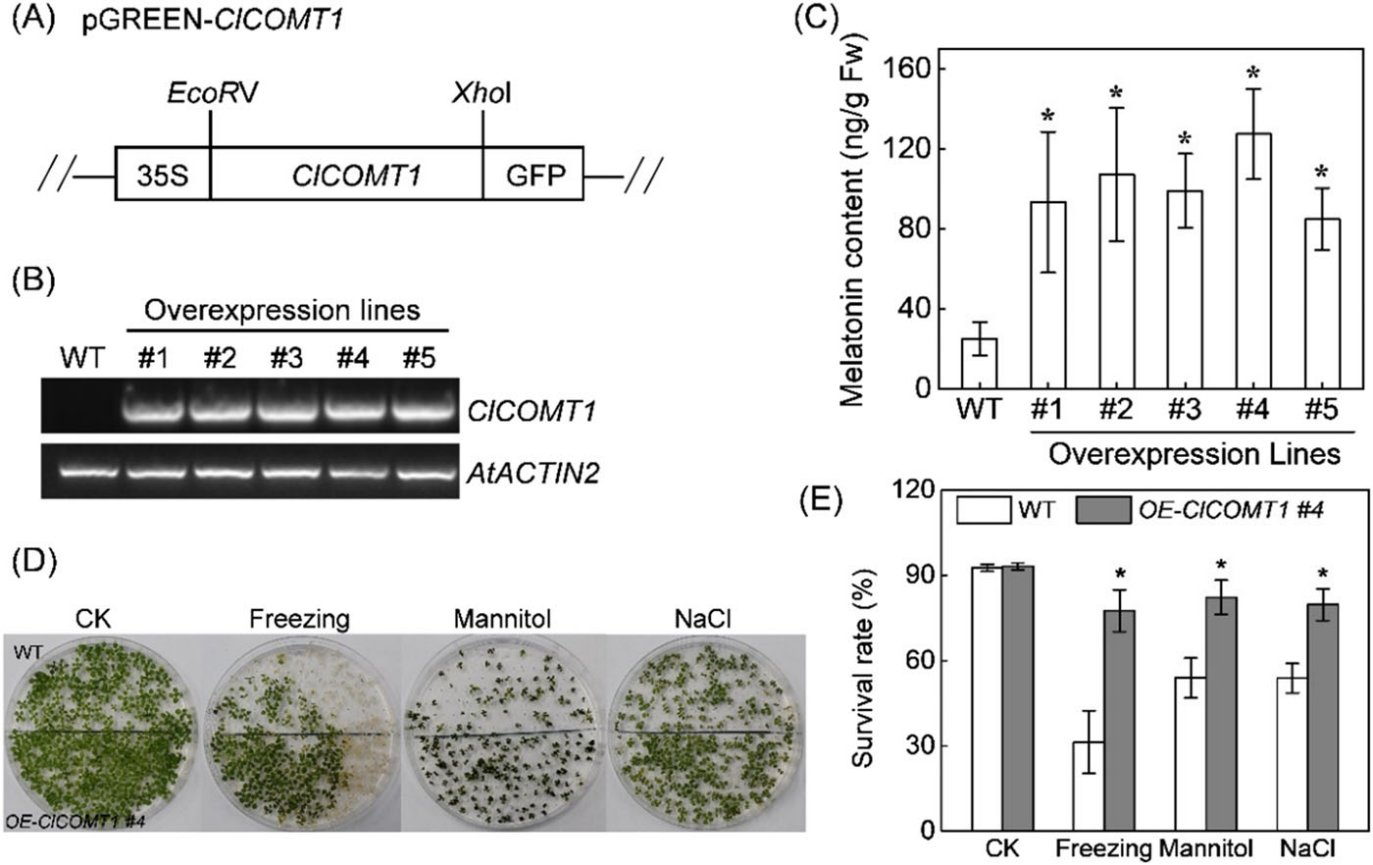
Effects of ClCOMT1 overexpression on melatonin contents and abiotic stress tolerance in Arabidopsis. **A** Schematic diagram of the pGREEN-*ClCOMT1* vector. **B** RT-PCR analysis of *ClCOMT1* expression in transgenic and wild-type (WT) *Arabidopsis*. **C** Melatonin contents in transgenic and WT *Arabidopsis*. **D**, **E** Phenotypes and survival rates of transgenic (line #4) and WT *Arabidopsis* after freezing, mannitol, or NaCl treatment. For freezing treatment, two-week-old *Arabidopsis* seedlings were exposed to −10 °C for 1 h, followed by recovery at 22 °C for 4 d. For drought and salt stress, *Arabidopsis* seeds were sown and grown on ½ MS containing 250 mM D-Mannitol and 75 mM NaCl, respectively. In (**C**, **E**), values are means ± SD (*n* = 3). Asterisk (*) indicates significant difference at *P* < 0.05. CK, control check; *ClCOMT*, *Citrullus lanatus caffeic acid O-methyltransferase*

To further examine whether *ClCOMT1* conferred plant tolerance to abiotic stresses, this gene was genetically transformed into *Arabidopsis* using a pGREEN-*ClCOMT1*-GFP vector (Fig. 8A). The expression of *ClCOMT1* was detected in the transgenic plants but not in wild-type plants (Fig. 8B). Melatonin contents in five transgenic lines were increased by 2.3- to 4.1-fold compared to that in the wild plants (Fig. 8C). Transgenic plants exhibited higher tolerance to cold, osmotic stress, and NaCl (Fig. 8D, E). For instance, the survival rates of transgenic plants (line #4) were 77.5%, 82.2%, and 79.6%, far higher than 31.3%, 53.9%, and 53.8% in wild plants after cold, mannitol, and NaCl treatment, respectively. These results indicated that *ClCOMT1* is a positive regulator of plant tolerance to abiotic stresses.

## Discussion

### *ClOMT03* (*Cla97C07G144540*) is a potential watermelon *COMT* gene (*ClCOMT1*)

The final step in melatonin biosynthesis is the conversion of NAS by proteins with ASMT activity. At present, *ASMT* genes have been cloned from rice, *Malus zumi*, *Arabidopsis*, and *Hypericum perforatum*^15,17–19^. However, *ASMT* genes are not conserved and thus are difficult to clone due to the lack of candidate genes in other plant species^15–17^, especially in those in which transformation is difficult, such as Cucurbitaceae. An alternative enzyme, COMT, also has ASMT activity and can methylate NAS into melatonin^20^. Surprisingly, the relative ASMT activity (*V*_*max*_*/K*_*m*_) of AtCOMT in melatonin synthesis was far higher than that of AtASMT both in vivo in *Arabidopsis* and in vitro^20^. In contrast, the substrate affinity (*K*_m_) of ASMT for NAS was higher than that of COMT, suggesting higher NAS affinity for COMT^20^. In addition to NAS, serotonin was also found to be catalyzed by COMT into 5-methoxytryptamine, which was converted into melatonin by SNAT^54^.

As *COMTs* belong to the *OMT* family, 16 putative *ClOMT* genes with an *O*-methyltransferase domain (PF00891) were identified in watermelon (Table 1)^22,55^. It is widely reported that melatonin exists in almost all plant tissues and that its production can be induced by various abiotic stresses, such as cold, drought, salinity, and excess heavy metals^6,53,56^. According to these previous results, bioinformatics analysis of *ClOMT*s was performed to screen candidate *ClCOMT* genes. *ClOMT03* (*Cla97C07G144540*) was considered a potential *ClCOMT* gene (renamed *ClCOMT1*) based on its high identities (60.00–74.93%) to known *COMT* genes involved in melatonin biosynthesis, constitutive expression in all tissues, and upregulation under abiotic stresses (Figs. 1, 2). In addition to the known COMT proteins^20,21^, ClCOMT1 was localized in the cytoplasm, where melatonin is synthesized from NAS^54^. In addition, the amino acid sequences and NAS-binding domains were relatively conserved among COMTs from dicotyledonous plants but not between COMTs from dicotyledonous and monocotyledonous species, suggesting that the *COMT* genes from dicotyledonous plants may originate from the same lineage (Fig. 3).

### *ClCOMT1* plays a direct role in melatonin biosynthesis in watermelon

Overexpression or knockout of *ClCOMT1* significantly increased or decreased melatonin contents, respectively, in the transgenic watermelon calli (Figs. 5, 6). Moreover, the melatonin contents were negatively correlated with the mutation rates of *ClCOMT1* in mutant watermelon calli (Fig. S1). These results indicated that *ClCOMT1* plays an essential role in melatonin synthesis in watermelon. In previous studies, *COMT* overexpression induced a slight increase in melatonin content in transgenic plants^21,30,57^. For instance, the *SlCOMT1* transcripts increased by 9-fold in transgenic tomato, whereas the melatonin content increased only by 35.2% compared to that in the nontransformed plants^57^. Interestingly, in watermelon calli with *ClCOMT1* overexpression, the increased levels of *ClCOMT1* expression (3.8- to 10.7-fold) were closely coupled with increases in melatonin contents, which were 2.2- to 7.0-fold relative to those in wild calli (Fig. 6). The difference in results may be attributed to the use of different tissues or species.

COMT is a multifunctional enzyme catalyzing the methylation of a diverse set of substrates such as 5-hydroxyferulic acid, caffeic acid, and quercetin^58^. The melatonin synthesis activity of COMT via NAS methylation is highly inhibited by the aforementioned substrates^20,21,59^. Owing to the ubiquitous presence of substrates that act as bottlenecks in the production of melatonin, ASMTs are required in plant species^21^. Here, we observed that transgenic watermelon calli with 100% mutation of *ClCOMT1* had 43.7% melatonin compared to wild calli (Fig. 5D). Therefore, it is plausible that there are *ClASMT* or other *ClCOMT* genes contributing to melatonin biosynthesis in watermelon. However, this speculation requires further investigation.

### *ClCOMT1* is a positive regulator of plant tolerance to abiotic stresses

Increasing studies have demonstrated that melatonin functions as an important regulator in plant adaptation to various environmental stresses. Application of melatonin at appropriate concentrations can enhance watermelon tolerance against cold, drought, and NaCl^31–33^. *SlCOMT1* overexpression enhanced salt tolerance in transgenic tomato plants^30,57^. Here, the expression of *ClCOMT1* was clearly induced, accompanied by increases in the accumulation of melatonin, after plants were challenged with cold, drought, and NaCl stress (Fig. 7). Furthermore, transgenic *Arabidopsis* plants with *ClCOMT1* expression showed higher tolerance to freezing, mannitol, and NaCl than the wild plants (Fig. 8). Taken together, *ClCOMT1* plays a positive role in regulating plant tolerance against abiotic stresses, and this role of *ClCOMT1* might be related to increased melatonin accumulation. In addition, COMT can methylate phenylpropanoid and flavonoid substrates and plays a key role in lignin biosynthesis^23^. In addition to melatonin, phenylpropanoids, flavonoids, and lignin play important roles in plant defense against various environmental stresses^60–63^. Therefore, *ClCOMT1*-induced abiotic stress tolerance might also be associated with altered phenylpropanoids, flavonoids, or lignin, but such speculation needs to be further investigated.

## Conclusion

As more functions of melatonin in plants have been confirmed, the key genes involved in melatonin synthesis should be explored in more plant species. In the current study, we reported a watermelon *COMT* gene (*ClCOMT1*) that plays an essential role in melatonin biosynthesis. We also confirmed that *ClCOMT1* is a positive regulator of plant tolerance to abiotic stresses, including cold, drought, and salinity. To our knowledge, *ClCOMT1* is the first melatonin biosynthetic gene cloned from a species in the Cucurbitaceae. It is likely that the findings presented here could be exploited to benefit cucurbit crop production in favorable and unfavorable environmental conditions.

## Supplementary information

Supporting information
