# Supplementary material for: The role of watermelon caffeic acid O-methyltransferase (ClCOMT1) in melatonin biosynthesis and abiotic stress tolerance
Source: Hortic Res. 2021 Oct 1;8:210. doi: 10.1038/s41438-021-00645-5 (PMC8484660; doi:10.1038/s41438-021-00645-5)
Supplement: Supplementary file 1 — Supporting information [file 41438_2021_645_MOESM1_ESM.docx]

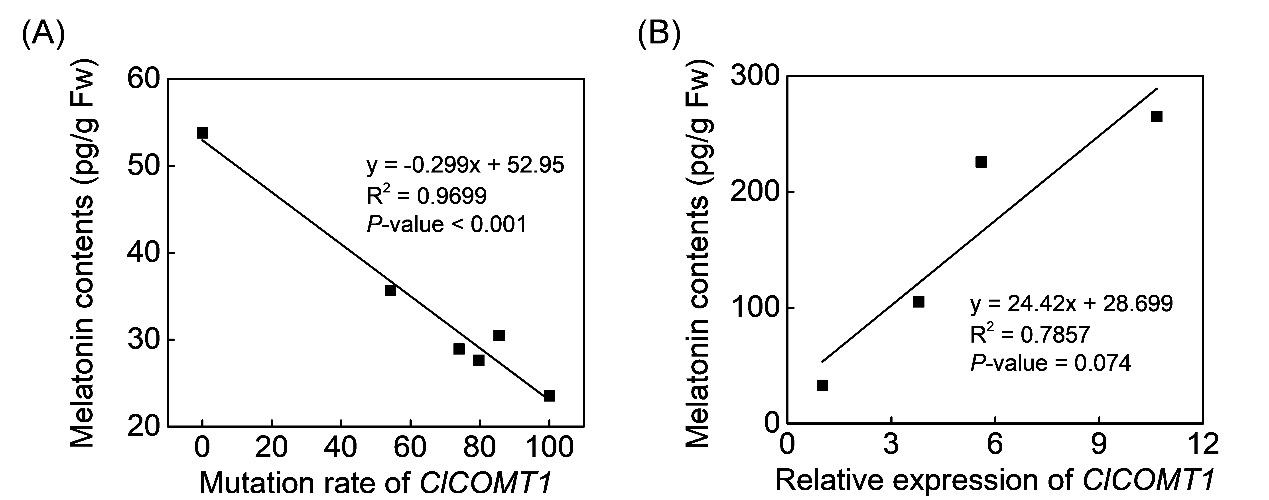


**Fig. S1** The correlation between melatonin contents and (**A**) mutation rates or (**B**) expressions of *ClCOMT1* in mutant or overexpressed watermelon calli, respectively*. ClCOMT*, *Citrullus lanatus* *caffeic acid O-methyltransferase*.


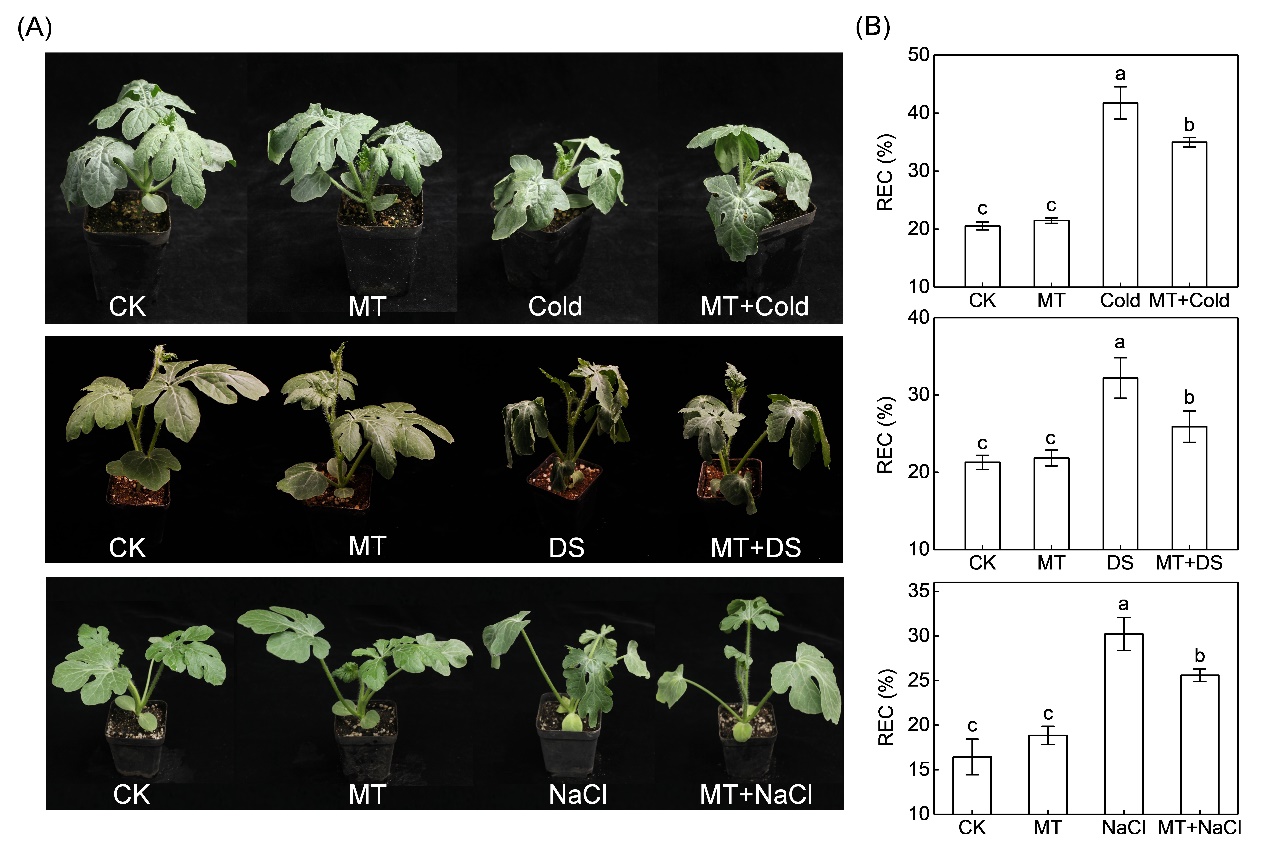


**Fig. S2** Effects of exogenous melatonin on watermelon tolerance to cold, drought (DS), and NaCl stress. Leaves of watermelon seedlings at the three-leaf stage were pretreated with 150 μM melatonin (MT). Twelve hours later, seedlings were exposed to cold at 4 ºC, DS, or NaCl stress as described in Figure 7. (**A**) Phenotypes were photographed and (**B**) relative electric conductivity (REC) was measured after cold, DS, or NaCl treatment for 48 h, 8 d, and 4 d respectively. In (**B**), values are means ± SD (*n* = 3). Means denoted with the same letter did not significantly differ at *P* < 0.05. CK, control check; *ClCOMT*, *Citrullus lanatus* *caffeic acid O-methyltransferase*.

**Table S1** Primers used for gene expression analysis and vector construction.

| Primer | Sequence (5’-3’) | Usage |
| --- | --- | --- |
| *β-actin*-F | CCATGTATGTTGCCATCCAG | RT-PCR for reference gene in watermelon |
| *β-actin*-R | GGATAGCATGGGGTAGAGCA |  |
| *ClCOMT1*-PCR-F | TCGCCACCAAGGGAGTCATTCA | RT-PCR for *ClCOMT1* |
| *ClCOMT1*-PCR-R | GCACAGCAGTGGACCTTGAAAC |  |
| pG-*ClCOMT1*-F | GAGGACAGCCCAAGCTACGCGTCTCGAGATGGGATCATCGGTGAGC | Subcellular localization analysis of ClCOMT1 protein |
| pG-*ClCOMT1*-R | ATCCCCCGGGCTGCAGGAATTCGATATCAGGTGTCTTGAGAAATTCC |  |
| *ClCOMT1*-oxjc-F | ATGGGATCATCGGTGAGC | Expression analysis of *ClCOMT1* in *Arabidopsis* |
| *ClCOMT1*-oxjc-R | AGGTGTCTTGAGAAATTCC |  |
| *AtACTIN2*-F | GGTAACATTGTGCTCAGTGGTGG | RT-PCR for reference gene in *Arabidopsis* |
| *AtACTIN2*-R | AACGACCTTAATCTTCATGCTGC |  |
| DT1-*ClCOMT1* | ATATATGGTCTCGATTGGGATCATCGGTGAGCGATGGTTTTAGAGCTAGAAATAGC | Construction of *ClCOMT1* knockout vector |
| DT2-*ClCOMT1* | ATTATTGGTCTCGAAACGCAAATCCAGCTCAATCGCCAATCTCTTAGTCGACTCTAC |  |
| HI-*ClCOMT1*-F | GGAGTGAGTACGGTGTGCGTACTATCCACTCCAATCCACT | Detection of mutant types |
| HI-*ClCOMT1*-R | GAGTTGGATGCTGGATGGGCTGGGAAGCGATTTGGGATG |  |
| 1305-*ClCOMT1*-F | ACGGGGGACTCTAGAGGATCCATGGGATCATCGGTGAGC | Construction of *ClCOMT1* overexpression vector |
| 1305-*ClCOMT1*-R | CTGGTCACCAATTCACACGTGCTAGTGGTGGTGGTGGTGGTGGAGGCGGAAG  AGGTTGTGTGTG |  |
